# Screening and prevention of ovarian cancer

**DOI:** 10.5694/mja2.52227

**Published:** 2024-02-14

**Authors:** Michail Sideris, Usha Menon, Ranjit Manchanda

**Affiliations:** 1 Wolfson Institute of Population Health, Queen Mary University of London, London, UK; 2 Institute of Clinical Trials and Methodology, University College London, London, UK; 3 Barts Health NHS Trust, London, UK; 4 London School of Hygiene and Tropical Medicine, London, UK

## Abstract

- Ovarian cancer remains the most lethal gynaecological malignancy with 314 000 cases and 207 000 deaths annually worldwide. Ovarian cancer cases and deaths are predicted to increase in Australia by 42% and 55% respectively by 2040.
- Earlier detection and significant downstaging of ovarian cancer have been demonstrated with multimodal screening in the largest randomised controlled trial of ovarian cancer screening in women at average population risk. However, none of the randomised trials have demonstrated a mortality benefit. Therefore, ovarian cancer screening is not currently recommended in women at average population risk. More frequent surveillance for ovarian cancer every three to four months in women at high risk has shown good performance characteristics and significant downstaging, but there is no available information on a survival benefit.
- Population testing offers an emerging novel strategy to identify women at high risk who can benefit from ovarian cancer prevention. Novel multicancer early detection biomarker, longitudinal multiple marker strategies, and new biomarkers are being investigated and evaluated for ovarian cancer screening.
- Risk-reducing salpingo-oophorectomy (RRSO) decreases ovarian cancer incidence and mortality and is recommended for women at over a 4–5% lifetime risk of ovarian cancer. Pre-menopausal women without contraindications to hormone replacement therapy (HRT) undergoing RRSO should be offered HRT until 51 years of age to minimise the detrimental consequences of premature menopause.
- Currently risk-reducing early salpingectomy and delayed oophorectomy (RRESDO) should only be offered to women at increased risk of ovarian cancer within the context of a research trial. Pre-menopausal early salpingectomy is associated with fewer menopausal symptoms and better sexual function than bilateral salpingo-oophorectomy.
- A Sectioning and Extensively Examining the Fimbria (SEE-FIM) protocol should be used for histopathological assessment in women at high risk of ovarian cancer who are undergoing surgical prevention.
- Opportunistic salpingectomy may be offered at routine gynaecological surgery to all women who have completed their family. Long term prospective opportunistic salpingectomy studies are needed to determine the effect size of ovarian cancer risk reduction and the impact on menopause.

Around 314 000 women worldwide are diagnosed with ovarian cancer annually and 207 000 women die of it.^1^ GLOBOCAN, the World Health Organization Global Cancer Observatory, predicts the number of ovarian cancer cases and deaths will rise globally by 36% and 47% respectively over 20 years.^2^ Correspondingly, the predicted increase in ovarian cancer cases and deaths in Australia is 42% and 55% respectively by 2040. Despite advances in treatment, ovarian cancer remains a lethal disease. High-grade serous ovarian cancer (HGSOC) is the commonest (> 80%) histological type. Around 80% of patients with ovarian cancer present in advanced stages III and IV, with five-year survival rates of 27% and 13% respectively.^3–5^ The principles of a screening program described over 50 years ago^6^ have subsequently been integrated into modern implementation frameworks, such as the Australian Population Based Screening Framework, to inform decision makers on key issues (criteria, principles of assessment, implementation, management) when assessing or considering screening programs in Australia.^7^ Ovarian cancer screening has been investigated in randomised controlled trials (RCTs) in women at average population risk and in cohort studies in women at high risk. These have included ultrasound, absolute cancer antigen 125 (Ca125), longitudinal Ca125, and multiple biomarker-based screening strategies. However, research so far has not led to the establishment of a national ovarian cancer screening program in Australia or elsewhere. Our improving ability to predict personalised ovarian cancer risk using complex modelling, the implementation of mainstreaming genetic testing at cancer diagnosis, and the unselected population-based approaches to identify women with moderate to high penetrance cancer susceptibility genes (CSGs) have broadened our ability to identify women at increased risk of ovarian cancer. This and the broad acceptance of the role of the fallopian tube in ovarian cancer etiopathogenesis have led to advances in preventive approaches to minimise ovarian cancer risk. These include broadening access for risk-reducing salpingo-oophorectomy (RRSO), along with implementing novel approaches such as risk-reducing early salpingectomy and delayed oophorectomy (RRESDO) in women at high risk, and opportunistic bilateral salpingectomy (OBS) in women at average population risk. Our review summarises and appraises the spectrum of ovarian cancer screening and targeted preventive approaches for reducing ovarian cancer risk.

## Methods

For this narrative review, we reviewed published literature using a combination of keywords, such as “ovarian cancer”, “screening”, “ovarian cancer screening”, “ovarian cancer prevention”, “prevention”, “novel screening biomarkers”, “risk-reducing surgery”, “risk-reducing salpingo-oophorectomy”, “risk-reducing early salpingectomy”, “risk-reducing early salpingectomy delayed oophorectomy”, “delayed oophorectomy”, and “opportunistic salpingectomy”. Searches were supplemented by manual review of references from relevant publications.

### Ovarian cancer screening in women at average population risk

The 2021 Cancer Australia position statement^8^ advises against ovarian cancer screening for women at average population risk using any tests, including pelvic examination, blood biomarkers, ultrasound or a combination of the above (Box 1). This was based on results from two large RCTs: the United Kingdom Collaborative Trial on Ovarian Cancer Screening (UKCTOCS)^3^ and the Prostate Lung Colorectal Ovarian (PLCO)^21^ cancer screening trial, which demonstrated no mortality benefit.

Overall, three RCTs have reported on ovarian cancer screening in women at average population risk.^3,21,22^ The Shizuoka Cohort Study of Ovarian Cancer Screening (SCSOCS)^22^ trial randomly assigned 82 487 post-menopausal women (screening arm, 41 688; control arm, 40 799) to annual screening with serum Ca125 interpreted using a 35 U/L single cut-off and ultrasound scan. It reported a non-significant stage shift (increase in early stage disease) between the screening (stage I ovarian cancer, 63%) and control arms (stage I ovarian cancer, 38%; *P* = 0.229).

In the PLCO-trial^21^ (screening arm, 39 105; control group, 39 111), annual screening was undertaken with serum Ca125 interpreted using a 35 U/L single cut-off and transvaginal ultrasound. There were 212 ovarian cancer cases and 118 ovarian cancer deaths in the screening group compared with 176 ovarian cancer cases and 100 ovarian cancer deaths in the control group (mortality rate ratio [RR], 1.18; 95% confidence interval [CI], 0.82–1.71). Screening identified only 28% of early stage ovarian cancers and there was no stage shift or mortality benefit.^21^

The UKCTOCS-trial^3^ randomly assigned (1:1:2) 202 638 post-menopausal women aged 50–74 years to annual multimodal screening (50 640), annual ultrasound scan screening (50 639), and controls (101 359). Multimodal screening used a sequential strategy with annual serum Ca125 and repeat Ca125 and transvaginal ultrasound as second line tests. Ca125 was interpreted using a longitudinal algorithm that incorporated age: the Risk of Ovarian Cancer Algorithm (ROCA). Compared with a single cut-off value, this approach doubled sensitivity while maintaining high specificity. The sensitivity and specificity of multimodal screening for detection of invasive epithelial ovarian cancer were 85.8% (95% CI, 79.3–90.9%) and 99.8% (95% CI, 99.8–99.8%) respectively.^23^

At a median follow-up of 16.3 years (interquartile range [IQR], 15.1–17.3 years), 2055 women were diagnosed with ovarian cancer (multimodal screening group, 522; ultrasound scan group, 517; controls, 1015). Screening was associated with a statistically significant stage shift, increased incidence of stage I–II disease (39.2%; 95% CI, 16.1–66.9%) and decreased incidence of stage III–IV disease (10.2%; 95% CI, -21.3% to 2.4%).^3^ Screening was also associated with a higher R0 surgical resection rate,^4^ an important clinical surrogate for better survival. However, ovarian cancer deaths between the arms did not differ (multimodal screening group, 296 deaths [*P* = 0.58]; ultrasound scan group, 291 deaths [*P* = 0.36]; controls, 619 deaths). Thus, screening did not reduce mortality. The reasons are likely multiple and include the effect size of the downstaging achieved, poor ovarian cancer biology, and availability of less effective treatments for ovarian cancer in 2001–2012. UKCTOCS highlights the importance of using “cancer mortality” rather than surrogate outcomes such as downstaging as the primary outcome in ovarian cancer screening trials. Any successful future screening strategy will likely need a much larger reduction in advanced stage disease and/or improvements in the lead and sojourn time than observed in UKCTOCS. In addition, clinicians and multidisciplinary teams will need to operate on a rising biomarker or abnormal screening result without radiological corroboration of any abnormality.

Besides ROCA, other longitudinal biomarker algorithms (eg, method of mean trends and parametric empirical Bayes) have been evaluated for ovarian cancer screening. These have been found to have comparable performance characteristics.^24,25^ Although other biomarkers have been evaluated along with Ca125 in ovarian cancer screening, these have not added significant value over Ca125 alone.^24^

### Ovarian cancer screening for women at high risk

Similarly, the Cancer Australia position statement on ovarian cancer screening does not recommend any form of screening for women at high risk and, hence, there is no national surveillance program for Australian women at high risk of ovarian cancer (Box 1).^8^ In the UK, the National Institute for Health and Care Excellence (NICE) is reviewing this issue within a guideline on familial ovarian cancer under development (GID-NG10225).^26^ Most of the evidence comes from prospective single-arm interventional trials in women who have a lifetime risk of ovarian cancer of 10% or more, and included predominantly *BRCA1* and *BRCA2* carriers, but also women with a strong family history of ovarian cancer. These trials include the UK Familial Ovarian Cancer Screening Study (UKFOCSS phase 1^27^ and phase 2^28^), the United States Cancer Genetics Network (CGN) and the Gynaecological Oncology Group (GOG) studies,^29,30^ and the Avoiding Late Diagnosis of Ovarian Cancer (ALDO) pilot trial.^31^

All these studies used a Ca125-based multimodal screening strategy, interpreted using the longitudinal Ca125 algorithm ROCA along with transvaginal ultrasound as a second line test. Annual screening using absolute serum Ca125 (single cut-off) and transvaginal ultrasound is not effective in women at high risk of ovarian cancer and is not recommended. This was evaluated in the UKFOCSS phase 1 study^27^ (*n* = 3563), in which only 31% of screen-detected cancers were early stage. However, it needs to be noted that screening was associated with significant delays in diagnosis. The UKFOCSS phase 2 study^28^ (4348 women; 84% *BRCA* carriers; 13 728 women screen-years) used a more frequent Ca125 ROCA-based screening strategy every four months. It demonstrated good sensitivity (94.7%; 95 CI%, 74.0–99.9%), specificity (98.9%; 95% CI, 98.7–99.1%), positive predictive value (10.8%; 95% CI, 6.5–16.5%) and negative predictive value (100%; 95% CI, 100–100%). Nineteen ovarian cancer cases were diagnosed during surveillance (13 cancers were screen-detected and six occult cancers were identified during RRSO following a normal screen). Screening was associated with a significant stage shift, with seven out of 19 cancers (36.8%) diagnosed within one year of the last screen, compared with 17 out of 18 cancers (94.4%) diagnosed more than one year after stopping screening, being stage IIIb–IV (*P* < 0.001). Furthermore, screen-detected cancers were more likely to have primary cytoreductive surgery with significantly lower use of neoadjuvant chemotherapy (1/19) compared with those presenting clinically (8/18; *P* = 0.008). Ovarian cancer treatment outcome studies indicate that primary cytoreductive surgery has better survival than interval surgery following neoadjuvant chemotherapy and is recommended in treatment guidelines. Similar performance characteristics from ROCA-based screening every three months were demonstrated in the US CGN/GOG trials (3692 women; 13 080 women screen-years).^29,30^ The UK ALDO pilot study evaluated ROCA-based surveillance every four months in 767 *BRCA1* and *BRCA2* carriers across 12 centres in the UK.^31^ ALDO demonstrated similar performance characteristics (sensitivity, specificity, negative predictive value, positive predictive value) of a Ca125 ROCA-based screening strategy in a real-world clinical setting to UKFOCSS phase 2, although the sample size and number of cancers detected were much smaller. Downstaging can lead to less radical surgery, with lesser morbidity and cost savings of poly (ADP-ribose) polymerase (PARP)-inhibitor treatment costs. The evidence from key trials in ovarian cancer screening for average and high risk populations is summarised in Box 2.^3,21,22,27–29,31,32^ Data on ovarian cancer screening for women with Lynch syndrome are limited, as the aforementioned trials mainly recruited *BRCA1* and *BRCA2* carriers. In addition, Lynch syndrome-related ovarian cancer is biologically different to *BRCA*-related HGSOC, being less aggressive and predominantly early stage at clinical diagnosis.^33^ Ovarian cancer screening in women with Lynch syndrome needs more research and is not currently recommended in Australia.^9^

Although findings from these trials on women at high risk appear encouraging, these are non-randomised and demonstration of a survival and mortality benefit is not possible. This makes drawing clear conclusions of benefit with respect to screening in women at high risk challenging. Screening is therefore not an alternative to risk-reducing surgery, which remains the mainstay of ovarian cancer prevention and risk management in women at high risk. However, there may be a potential role as an interim risk management strategy in women delaying risk-reducing surgery following careful counselling, as an option compared with symptom awareness alone. These issues are under review by a NICE Guideline Committee, with a draft guideline in preparation.^34^ A summary of published guideline recommendations regarding ovarian cancer screening in women at average population risk and at high risk is presented in Box 1.

### Novel biomarkers and ovarian cancer screening strategies

Several novel biomarkers and screening strategies are currently being investigated globally and may hold promise for the future. Examples include DNA methylation biomarkers (eg, Women’s Risk Identification for Ovarian Cancer [WID-OC] Index),^35^ cell-free DNA,^36^ circulating tumour DNA (ctDNA),^37,38^ glycosylated Ca125,^39^ Olink biomarkers,^40^ other novel biomarkers,^41^ use of multimarker longitudinal algorithms,^24^ and multicancer early detection (MCED) biomarker strategies.^42^ MCED uses next-generation genetic and epigenetic technologies involving a range of potential platforms to screen for multiple cancer types at the same time. Screening strategies using MCED tests use second line positron emission tomography–computed tomography (PET-CT),^43^ along with DNA patterns to identify cancer site^44^ and machine learning algorithms to improve performance.^45^ A number of MCED studies are investigating a range of biomarkers and tests for pan-cancer early detection. A particular challenge is the low prevalence of most cancer types in populations at average risk (Box 3).^38,44,45^ Although the MCED approach is associated with better aggregate sensitivity or positive predictive value for multiple cancers, the accuracy or performance for individual cancer types varies and may be limited. Moreover, sensitivity for early stage disease, in particular early stage ovarian cancer, remains limited.^46^ Other challenges that need resolving include accuracy of tissue of origin; testing pathway and strategy, including frequency, triage and follow-up; level of over diagnosis; and impact on mortality and cost-effectiveness.

It is important for the adoption of an effective general population ovarian cancer screening strategy that screening trials have mortality as a primary outcome, given that surrogate markers such as downstaging or R0 resection rate have not proved reliable in predicting mortality reduction.

Studies have evaluated multiple biomarkers in addition to Ca125 in pre-diagnostic samples of women developing ovarian cancer, but these additional markers did not improve screening performance compared with Ca125 alone.^24,47^ A longitudinal multiple biomarker-based early diagnosis strategy may identify cancers missed by Ca125 alone and, therefore, earlier than routine clinical detection, or may detect ovarian cancer before detection by Ca125 and thereby enable a greater reduction in incidence of late stage disease.^48^ Some new biomarkers with potential include glycovariants of Ca125,^49^ Copenhagen Index,^50^ Olink panel,^51^ and long interspersed nuclear element (LINE-1) open reading frame 1 protein (ORF1p)^52^ biomarkers. There is growing consensus that most HGSOC cancers arise from serous tubular intraepithelial carcinoma (STIC) lesions in the fallopian tubes. Modelling studies suggest a fallopian tube STIC lesion takes six to seven years to develop into invasive ovarian cancer.^53^ A validated STIC biomarker is urgently needed and may lead to huge strides in the quest for an effective ovarian cancer screening strategy.

### Improving identification of women at increased risk of ovarian cancer

Women with one first degree relative have an estimated 2.96 familial relative risk (95% CI, 2.35–3.72) of developing ovarian cancer,^54^ while women with two or three first degree relatives with ovarian cancer have significantly higher familial relative risk. In addition to the traditional *BRCA1* and *BRCA2* CSGs, a number of newer moderate penetrance ovarian cancer CSGs (*RAD51C, RAD51D, BRIP1, PALB2*), with lifetime ovarian cancer risks ranging from 5% to 13%, have been identified and are now routinely tested along with mismatch repair (Lynch syndrome) genes. Surgical prevention to minimise ovarian cancer risk is now recommended and offered to these CSG carriers.^55,56^ Around 15–22% of ovarian cancers in the general population and 40% in the Jewish population are caused by ovarian cancer CSGs and are thus potentially preventable.

Maximising identification of women at increased ovarian cancer risk who can benefit from targeted preventive interventions is essential to reduce future burden of disease. In the UK, Australia and in other health systems, individuals are offered genetic testing dependent on fulfilling established clinical or family history-based criteria.^8^ A probability of 10% or greater of carrying a *BRCA* pathogenic variant is the current clinical threshold for being offered genetic testing. This approach is plagued by restricted access and underutilisation of testing. Importantly, an estimated 50–80% of pathogenic variant carriers for these ovarian cancer CSGs do not fulfil current clinical genetic testing criteria,^57–59^ and 97% of individuals carrying high penetrance monogenic CSGs (eg, *BRCA* carriers) remain unidentified despite 25 years of criteria-driven genetic testing.^60^

Offering unselected genetic testing at cancer diagnosis increases the identification of individuals carrying CSGs who can benefit from secondary cancer prevention, along with identification through cascade testing of unaffected family members who can opt for primary cancer prevention. This is now recommended in guidelines and available for ovarian, endometrial and bowel cancers.^57,61–63^ It is likely that this will become available for all individuals with breast cancer and other solid cancers in the future.^64,65^

However, why should we wait for someone to develop cancer in order to identify people in whom we can prevent cancer? Altering this paradigm to unselected population testing can address these limitations and provides a strategy to maximise prevention. The largest evidence base for population testing currently comes from *BRCA* testing studies in the Jewish population. Jewish population-based *BRCA* testing more than doubles the number of *BRCA* carriers identified; is safe, feasible and acceptable; has high satisfaction; reduces anxiety; does not detrimentally affect quality of life or lifestyle; and is cost-saving for the health system.^58,66–69^ As a result, population-based *BRCA* testing was introduced in Israel in 2022^70^ and was recently launched as a pilot National Health Service program for the UK Jewish population in February 2023.^71^

Sophisticated ovarian cancer risk models incorporating multiple risk factors, including a polygenic risk score (PRS), family history, and epidemiological, hormonal and reproductive factors, have recently been validated.^72^ These can be used to predict a personalised ovarian cancer risk. Women with ovarian cancer risk greater than 4–5% can be offered surgical prevention.^56,73^ The PROMISE pilot study on population genetic testing showed acceptability, feasibility, high satisfaction and reduced cancer worry with personalised ovarian cancer risk prediction.^74^ Population-based testing for ovarian cancer CSGs has been shown to be cost-effective for the health systems in the UK, the US, Australia, the Netherlands, China and Brazil.^75–77^

Finding unaffected people at increased ovarian cancer risk in the general population, including pathogenic variant carriers for monogenic ovarian cancer CSGs, should be an urgent priority for population genomics and health systems. Prospective general population-based genetic testing trials are being pioneered in the UK and Australia to identify individuals at increased cancer risk for targeted screening and prevention.^78^ The PROTECT-C study^79,80^ is evaluating the impact of panel genetic testing for nine medically actionable (*BRCA1, BRCA2, PALB2, RAD51C, RAD51D, BRIP1, MLH1, MSH2, MSH6*) hereditary breast and ovarian cancer and Lynch syndrome genes, along with concurrent personalised breast cancer and ovarian cancer risk prediction using PRS and epidemiological and reproductive factors in unselected UK women aged over 18 years. The Australian DNA Screen study^81^ is offering routine genetic testing for medically actionable hereditary breast and ovarian cancer, Lynch syndrome and familial hypercholesterolaemia genes (*BRCA1, BRCA2, MLH1, MSH2, MSH6, PMS2, LDLR, APOB, PCSK9*) to 18–40-year-old individuals.^81^ Outcomes from these programs will help inform future policy and guidelines related to population genetic testing.

### Surgical prevention for women at increased risk of ovarian cancer

#### Risk-reducing salpingo-oophorectomy

Surgical prevention remains the gold standard and most effective ovarian cancer prevention strategy. The 2011 National Breast and Ovarian Cancer Centre and Cancer Australia guidelines currently recommend RRSO for women in Australia who are *BRCA1* or *BRCA2* carriers and/or have Lynch syndrome.^8,9,82^ RRSO with peritoneal washings is undertaken through minimal access surgery and is associated with minimal surgical morbidity.^83^ RRSO can reduce ovarian cancer risk by 80–97% in *BRCA* carriers,^84^ reduce ovarian cancer and all-cause mortality,^84^ reduce ovarian cancer risk by 94% in women at the average population-level risk,^85^ and systematic reviews show it is cost-effective.^86^ A small residual risk (2–4%) of primary peritoneal cancer was reported in *BRCA* carriers,^84^ but recent reports highlight negligible residual risk levels.^87^ RRSO is cost-effective at 4–5% or more lifetime ovarian cancer risk thresholds.^73,88^ This saves seven to ten years of a woman’s life and has been found to be acceptable.^73,88,89^ This 4–5% lifetime ovarian cancer risk threshold has been supported by a Royal College of Obstetricians and Gynaecologists scientific impact paper^56^ and a UK Cancer Genetics Group consensus statement.^55^ An American group suggested a lower 3–4% lifetime ovarian cancer risk threshold be considered.^90^ This approach broadens access to surgical prevention for more women at increased risk of ovarian cancer.

It is critical that a standardised histopathological Sectioning and Extensively Examining the Fimbria (SEE-FIM) protocol be used for histological examination after RRSO. Around 5.1% of cases (95% CI, 1.9–10.83%) may have occult STIC or invasive cancer at histology,^83^ with most lesions (70%) being fimbrial or distal tubular and more likely to be missed without a SEE-FIM protocol.^91^ Surgeons should be aware that avoiding thermal injury to the tubal ends doubles the identification of occult tubal lesions.^92^ STIC and invasive cancers identified should be referred to a tertiary gynaecological oncology centre for multidisciplinary team management.

RRSO in pre-menopausal women leads to early surgical menopause, which can detrimentally affect short and long term health outcomes. Pre-menopausal RRSO is associated with increased risks of osteoporosis, neurocognitive decline, heart disease, sexual dysfunction, drop in libido, and vaginal dryness.^93^ These can be minimised or ameliorated (but not necessarily eradicated) by HRT. Symptomatology, particularly sexual dysfunction, is poorer compared with women who have not undergone pre-menopausal oophorectomy. Although overall satisfaction levels remain high (88–95%), pre-menopausal RRSO is also associated with much higher (~9%) regret than post-menopausal (~1%) RRSO.^94^ HRT is recommended following pre-menopausal RRSO in women aged up to 51 years provided there is no other contraindication.^56^ Access to and compliance with HRT is therefore an important issue in the long term follow-up of these women. Poor access, with at times low and varying uptake rates have been reported in the past. Higher uptake rates are reported by women managed in specialist services and high risk clinics.^94,95^ For women who had RRSO alone (intact uterus), combined oestrogen and progestogen HRT is the standard recommendation. Women with Lynch syndrome may also undergo a hysterectomy due to increased endometrial cancer risk and they therefore need oestrogen-only HRT. HRT can be commenced immediately post-operatively and transdermal preparations have a better side-effect profile. More detailed descriptions on HRT and non-HRT management for early menopause can be found elsewhere.^56^

#### Risk-reducing early salpingectomy and delayed oophorectomy

The broad acceptance of the role of the fallopian tube in the aetiopathogenesis of HGSOC, coupled with the detrimental consequences of early menopause, has enabled the attractive proposition of an alternative two-stage surgical prevention option, with pre-menopausal early salpingectomy (first step) followed by delayed oophorectomy nearer or at menopause. This enables women who may not have undergone RRSO to retain ovarian function for longer while obtaining a level of ovarian cancer risk reduction. The precise level of ovarian cancer risk reduction is not yet established and the long term impact on ovarian function is unknown. RRESDO is currently being offered within research trials in the UK, the US and the Netherlands. These studies have evaluated acceptability, safety, quality of life, impact on menopause, and cost-effectiveness of this approach. The PROTECTOR trial compares RRESDO, RRSO and no surgery (controls) across 42 UK centres.^96^ Preliminary results from the Dutch multicentre TUBA trial show RRESDO is an acceptable alternative for patients and clinicians,^94,95^ and is associated with better sexual function and fewer menopause symptoms than RRSO.^97^ RRESDO should currently only be offered within a research study setting.

### Surgical prevention options for average risk individuals

Widespread recognition of the contribution of the fallopian tube to ovarian cancer aetiology has led to an increasing uptake of OBS as a method of ovarian cancer prevention among women undergoing routine benign gynaecological surgery, such as hysterectomy and sterilisation. Available data confirm OBS is safe, takes minimal additional time, does not increase complication rate, and has acceptable morbidity, albeit a potentially increased need for analgesia.^98^ A higher risk of haemorrhage (odds ratio [OR], 1.24; 95% CI, 1.15–1.33) and blood transfusion may occur in women undergoing salpingectomy during a caesarean delivery, although the absolute effect size (3.8% *v* 3.1%) is small.^99^ OBS is frequently discussed or offered in Australia, usually at the time of hysterectomy.^100^

Initial evidence for ovarian cancer risk reduction with salpingectomy comes from large Scandinavian population-based case–control studies from Denmark (ovarian cancer cases, 13 241; 1:15 age-matched controls)^101^ and Sweden (salpingectomy cases, 34 433; controls, 5449919).^102^ These studies suggested bilateral salpingectomy was associated with a 42% (OR, 0.58; 95% CI, 0.36–0.95)^101^ and 65% (hazard ratio [HR], 0.35; 95% CI, 0.17–0.73)^102^ reduction in ovarian cancer risk. These studies have been criticised for being retrospective, not correcting for all confounders, having indication and detection biases, having a small number of events, the intervention not being an opportunistic salpingectomy, and the comparator not being the routine gynaecological surgical procedure. Systematic reviews suggest poor quality of evidence from these studies. A lower level of ovarian cancer risk reduction (HR, 0.72; 95% CI, 0.56–0.93) was found from bilateral salpingectomy in a follow-up study after correcting for pelvic inflammatory disease.^103^ Recent emerging prospective data confirm reduction in serous ovarian cancer risk in particular.^104^ Although short term follow-up data for up to a couple of years are reassuring, these are not predictive of onset of menopause, and the impact on long term endocrine function and menopause remains to be elucidated.

Hence, initial published data from OBS are helpful and reassuring. However, there is a need for prospective long term high quality studies to inform outcomes of ovarian cancer risk reduction and endocrine function for informed counselling and recommendations for clinical practice.

## Conclusions

Some important practice points are listed in Box 4. Ovarian cancer screening is not currently recommended for women at population-level risk in Australia or elsewhere, and a survival benefit has not yet been demonstrated in women at high risk. Surrogate markers such as stage shift seen in the UKCTOCS trial are not reliable endpoints for ovarian cancer screening trials. RRSO is the most clinically effective method for preventing ovarian cancer. There is broadening access to RRSO with it also being recommended for intermediate risk ovarian cancer CSGs and individuals with more than 4–5% lifetime risk of ovarian cancer. Early salpingectomy is associated with fewer menopause symptoms and better sexual function, but the impact on the level of ovarian cancer risk reduction and long term menopause remains to be established. RRESDO should currently only be offered in research studies. Greater unselected testing at cancer diagnosis and upcoming population-based testing strategies can provide a pivotal change to optimise identification of individuals at increased risk of ovarian cancer who can benefit from preventive strategies.

## 1 Summary of internationally published guidelines on ovarian cancer screening

**Table:**

Society (country, year)	Recommendations (average risk individuals)	Recommendations (high risk individuals)
Cancer Australia* (Australia, 2019 [updated 2021])^8,9^	No role of screening for ovarian cancer No evidence that asymptomatic women benefit from any test, including Ca125, transvaginal ultrasound, clinical examination or other biomarkers	Insufficient evidence to support the role of screening for ovarian cancer No evidence that asymptomatic women benefit from any test, including Ca125, transvaginal ultrasound, clinical examination or other biomarkers
British Gynaecological Cancer Society (UK, 2017)^10^	There is no role for screening programs for ovarian cancer Screening is currently not recommended	The role of ovarian cancer screening in women at high risk is yet to be established. Screening outside a research trial not yet recommended
Society of Gynaecological Oncology^11^ and Ovarian Cancer Alliance^†^ (US, 2023)^12^	Screening the general public for ovarian cancer using Ca125 and ultrasound does not save lives in the general population; and population screening, which can reduce mortality, is likely decades away Screening women at average population risk is not recommended	“While the gold standard of care for women at high risk of ovarian cancer is preventative surgery, screening can be a reasonable option for those who have had thorough counselling and have made an informed decision to delay surgery or are unwilling to undergo surgery”
European Society of Medical Oncology (EU, 2023)^13^	na	Ultrasound and serum Ca125 every 6 months from the age at which risk-reducing surgery is recommended until completed
United States Preventive Services Task Force (USPSTF) (US, 2018)^14^	The USPSTF does not recommend routine screening for ovarian cancer using any method in asymptomatic women at average population risk	na
American College of Obstetricians and Gynaecologists and Society of Gynaecological Oncology (US, 2017)^15^	No role of screening in average risk women	“Routine ovarian cancer screening with Ca125 or transvaginal ultrasound is not recommended. Ca125 or transvaginal ultrasound may be reasonable for short term surveillance in women at high risk of ovarian cancer starting at age 30–35 years until the time they choose to pursue risk-reducing bilateral salpingo- oophorectomy, which is the only proven intervention to reduce ovarian cancer-specific mortality”^16^
Scottish Intercollegiate Guidelines Network (Scotland, 2018)^17^	Screening for ovarian cancer in the general population should not be performed outside the research setting	“Screening for ovarian cancer in high risk groups should only be offered in the context of a research study”
National Comprehensive Cancer Network (US, 2021)^18,19^	Ovarian cancer screening is not recommended	For patients who have not elected to have RRSO, transvaginal ultrasound and Ca125 for ovarian cancer screening although of uncertain benefit may be considered at the clinician’s discretion
British Columbia Genital Tract Cancers in Females Guidelines (Canada, 2014)^20^	Ovarian cancer screening is not recommended	Ovarian cancer screening is not recommended

Ca125 = cancer antigen 125; EU = European Union; na = not applicable; RRSO = risk-reducing salpingo-oophorectomy; UK = United Kingdom; US = United States.

* Statement endorsed by the Australian Society of Gynaecological Oncologists, the Royal Australian and New Zealand College of Obstetricians and Gynaecologists, Ovarian Cancer Australia, the Royal College of Pathologists of Australasia, the Australian College of Rural and Remote Medicine, and Cancer Council.

† Joint statement.

## 2 Screening average and populations at high risk for ovarian cancer (evidence from key trials)

**Table:**

	Trial design/arms	Key findings and conclusion
**Populations at average risk**		
SCSOCS (Japan, 1985–1999)^22^	Multicentre RCT Participants: 82 487 asymptomatic post-menopausal women (median age, 58 years) Screening strategy: combined annual serum Ca125 and transvaginal ultrasound Follow-up: mean 9.2 years (range, 3–14 years) Ovarian cancers: 27/41 688 in the screened arm and 32/40 779 in the control arm	Stage: statistically non-significant increase in stage I cancers between the screening and control arms (63% *v* 38% respectively;*P* = 0.2285); 9 screen-detected stage II–IV cancers *v* 18 in the control arm Ovarian cancer mortality: not reported Screening did not result in downstaging or reduction in ovarian cancer mortality
PLCO (US, 1993–2001)^21^	Multicentre RCT Participants: 78 216 post-menopausal women (range, 55–74 years) Screening strategy: combined annual serum Ca125 (6 years) and transvaginal ultrasound (4 years) Follow-up: median 12.4 years (IQR, 10.9–13.0 years) Ovarian cancers: 212/39 105 in the screened arm and 176/39 111 in the control arm	Stage: stage III–IV ovarian cancer cases were similar in the screen and control arms (77% *v* 78% respectively) Ovarian cancer mortality: 3.1 deaths per 10 000 person-years in the screening arm *v* 2.6 in the control arm (RR, 1.18; 95% CI, 0.82–1.71) Screening did not result in downstaging or reduction in ovarian cancer mortality
UKCTOCS (UK, 2001–2020)^3^	Multicentre, multi-arm RCT with 1:1:2 randomisation to the two screening and the no screening arms respectively Participants: 202 638 post-menopausal women (median age, 63 years; range, 50–74 years) Screening strategy: multimodal (longitudinal serum Ca125 interpreted using the ROCA calculation with transvaginal ultrasound and repeat Ca125 as a second line test) and transvaginal ultrasound as first and second line tests Follow-up: median, 16.3 years (IQR, 15.1–17.3 years) Ovarian cancers: 522/50 625 in the multimodal screening arm; 517/50 623 in the ultrasound scan arm; 1016/101314 in the control arm	Stage: multimodal screening *v* no screening — reduction in the incidence of stage IV disease by 24.5% (95% CI, -41.8 to -2.0) and increase in stage I by 47.2% (95% CI, 19.7–81.1). Ultrasound scan *v* no screening: no difference in stage Ovarian cancer mortality: no significant reduction in ovarian and tubal cancer deaths compared with no screening in the multimodal screening (*P* = 0.58) or ultrasound scan (*P* = 0.36) groups Screening resulted in downstaging of ovarian cancer in the multimodal screening arm but there was no reduction in ovarian cancer mortality
MD Anderson (US, 2001–2011)^32^	Single-centre single-arm pilot trial Participants: 4051 post-menopausal low risk women (median age, 59 years; range, 50–74 years) Screening strategy: multimodal screening as described for UKCTOCS Follow-up: 16 832 screen-years Ovarian cancers: 4	Stage: 3/4 participants were stage I, 1/4 were stage II Ovarian cancer mortality: not an outcome measure as pilot trial Performance characteristics: specificity, 99.9% (95% CI, 99.7–100%); PPV, 40% (95% CI, 12.2–73.8%) Multimodal screening is feasible and has high specificity and PPV
**Populations at high risk**		
UKFOCSS phase 1 (UK, 2002–2008)^27^	Single-arm interventional trial Participants: 3563 women with ≥ 10% lifetime risk of ovarian cancer Screening strategy: annual serum Ca125 interpreted using single cut-off and transvaginal ultrasound Follow-up: mean, 3.2 years per woman WSYs: 11 366 Ovarian cancers: 26 cases during 11233 screen-years, additional 10 ovarian cancers beyond censoring 365 days after last screen	Performance characteristics for ovarian cancer detection within 1 year of annual screen Sensitivity: 81.3% (95% CI, 54.3–96.0) or 87.5% (95% CI, 61.7–98.5) depending on whether occult ovarian cancers were classified as false negatives or true positives respectively Stage: 31% of cancers were early stage I-II Annual screening with annual Ca125 should not be used in populations at high risk
GOG/CGN (US, 2001–2011)^29^	Single-arm interventional trial Participants: 3692 women with strong family history or *BRCA1* and *BRCA2* pathogenic variant Screening strategy: ROCA-based multimodal screening every 3 months Follow-up: median, 6 years WSYs: 13 080 Ovarian cancers: 19 cancers (4 screen-detected at prevalence, 6 at incidence screen, 9 occult at RRSO, and 1 screen negative)	Performance outcomes reported: specificity and PPV compared with baseline general population screening values (90% and 10% respectively); specificity for ultrasound referral was 92% *v* 90% (*P* = 0.0001); PPV was 4.6% *v* 10% (*P* >0.10) Early stage: 3/6 incident ovarian cancer cases (50%; 95% CI, 12–88%) ROCA-based screening every 3 months had better early stage disease sensitivity (50%) at high specificity
UKFOCSS phase 2 (UK, 2007–2012)^28^	Single arm interventional trial Participants: 4348 high risk women at ≥10% lifetime risk of ovarian cancer (84% *BRCA* carriers) Screening strategy: ROCA-based multimodal screening every 4 months Follow-up: median, 4.8 years WSYs: 13 728 Ovarian cancers: 19 cases ≤ 1 year of end of screening; 18 cases > 1 year after screening stopped (these groups were compared)	Performance characteristics for detection of ovarian cancer within one year of screening using modelling: sensitivity, 94.7% (95% CI, 74.0–99.9%); PPV, 10.8% (95% CI, 6.5–16.5%); NPV, 100% Stage IIIb–IV: 7/19 cancers (36.8%; 95% CI, 16.3–61.6%) diagnosed within 1 year of end of screening 17/18 cancers (94.4%; 95 CI%, 72.7–99.9%) diagnosed >1 year after screening stopped, indicating a significant stage shift (*P* < 0.001) Other outcomes: significantly lower use of neoadjuvant chemotherapy in the cancers diagnosed during screening *v* the ones diagnosed during surveillance 1/19 (5.3%; 95% CI, 0.1–26.0%) *v* 8/18 (44.4%; 95% CI, 21.5–69.2%; *P* =0.008), and 18/19 (94.8%; 95% CI, 74.0–99.9%) cancers diagnosed during screening had optimal cytoreduction (R0) Screening every 4 months can result in significant downstaging, but given the trial design, it is not possible to estimate the impact on ovarian cancer mortality	
ALDO (UK, 2018–2019)^31^	Single-arm interventional trial Participants: 875 *BRCA1* and *BRCA2* pathogenic variant carriers who were not willing to undergo RRSO Screening strategy: serum Ca125 every 4 months using ROCA-based multimodal strategy Follow-up: median, 1.9 WSYs per woman (range, 0.04-2.72 WSYs) WSYs: 1277 Ovarian cancers: 8 in the screened arm (2 occult, 6 screen-detected)	Performance for ovarian cancer detection at 4 months after the last surveillance: sensitivity, 87.5% (95% CI, 47.3–99.7%); specificity, 99.9% (95% CI, 99.9–100%); PPV, 75% (95% CI, 34.9–96.8%); NPV, 99.9% (95% CI, 99.9–100%) Stage: 3/6 screen-detected ovarian cancers were stage Illa or below Validated the performance characteristics obtained in UKFOCSS Screening may be cost-saving due to lower PARP inhibitor costs

ALDO = Avoiding Late Diagnosis of Ovarian Cancer; Ca125 = cancer antigen 125; CI = confidence interval; GOG/CGN = Gynaecological Oncology Group and Cancer Genetics Network; IQR = interquartile range; NPV = negative predictive value; PARP = poly (ADP-ribose) polymerase; PLCO = prostate, lung, colorectal and ovarian; PPV = positive predictive value; RCT = randomised controlled trial; ROCA = Risk of Ovarian Cancer Algorithm; RR = relative risk; SCSOCS = Shizuoka Cohort Study of Ovarian Cancer Screening; UK = United Kingdom; UKCTOCS = United Kingdom Collaborative Trial on Ovarian Cancer Screening; UKFOCSS = UK Familial Ovarian Cancer Screening Study; US = United States; WSYs = women screen years.

## 3 Summary of multicancer early detection (MCED) trials and cohort studies

**Table:**

Trial (country)	Trial design	Key findings
DETECT A (US)^*^,^38^	Multicentre single arm interventional trial; 10 006 women (65–75 years old) from 18 clinical sites with no previous history of cancer MCED biomarker: blood test to detect early version of a multi-analyte (DNA and protein) MCED test (CancerSEEK) combined with PET-CT, without use of machine learning Primary outcomes: feasibility and safety	CancerSEEK detected 26/96 (27%) cancers; 15 of these 26 underwent PET-CT and 9/15 were surgically excised; 5/26 were stage I and 3/26 were stage II 24 cancers were detected by standard-of-care screening; 46 cancers were identified by neither approach^43^ False positive results led to 1.0% of participants undergoing PET-CT imaging; 0.22% of participants underwent a futile invasive diagnostic procedure CancerSEEK can be a safe adjunct to standard of care screening
The Circulating Cell-free Genome Atlas Study (CCGA)* (US; data from ClinicalTrials.gov Identifier NCT0288978)^45^	Substudy from CCGA (prospective, multicentre, case–control, observational study with longitudinal follow-up) The pre-specified independent validation set consisted of 4077 participants, 1254 controls, 2823 cancers at one-year follow-up Test and validation set for analysis MCED biomarker: blood test measuring cfDNA after bisulfite sequencing to target > 100 000 informative methylation regions, with development and validation of a classifier for cancer detection and tissue origin Primary outcome: performance of targeted methylation analysis of cfDNA to detect and localise multiple cancers across all stages in the validation set	Any cancer signal detection: specificity, 99.5% (95% CI, 99.0–99.8%); sensitivity, 51.5% (95% CI, 49.6–53.3%) For ovarian cancer detection: sensitivity all stages, 83.1% (95% CI, 72.2–90.3%); sensitivity stage I, 50% (95% CI, 23.7–76.3%) MCED could be used as an adjunct to existing screening policies
PATHFINDER (US; ClinicalTrials.gov Identifier NCT04241796)^44^	Multicentre, prospective, longitudinal, interventional multicentre study 6662 participants (≥ 50 years old) assigned to either cohort A (no cancer risk factors) or cohort B (≥ 1/3 pre-defined risk factors) MCED biomarker: blood test focused on targeted methylation plasma cfDNA-based named “Galleri” (early version MCED-E and later version MCED-Scr) Primary outcome: number and types of subsequent diagnostic tests, as well as time needed for diagnostic resolution after a signal is identified by the test	Cancer signal was detected in 92 participants MCED detected 35 cancers; 48% of them were stage I or II 71% of detected cancers are types of cancers that do not qualify for routine screening PPV for MCED-E and MCED-Scr: 38% and 43.1% respectively; > 1% false positive rates for both versions Accuracy of cancer signal prediction (CSO): 97%
Ongoing MCED trials/cohort studies		
DOvEEgene phase 3^†^ (Canada; ClinicalTrials.gov Identifier NCT04891029)	Two-centre, case–control, cross sectional trial Target population: 3600 women aged 45–70 years MCED biomarker: uterine Pap smear (DOvEEgene) to detect cfDNA using somatic cancer-related mutations, in combination with an established machine learning algorithm	Trial is ongoing Primary outcome: performance of detection of ovarian and endometrial cancer
NHS Galleri trial^‡^ (UK; ClinicalTrials.gov Identifier NCT05611632)	Pragmatic randomised controlled trial with 1:1 randomisation Participants: 140 000 individuals, aged 50–77years, who have not received previous cancer treatment for the past 3 years. MCED biomarker: blood test (Galleri) using DNA methylation patterns to measure cfDNA and assign tissue of origin	Recruitment is complete (31 August 2021 – 16 July 2022) Primary outcome: incidence rate of stage III–IV cancers adjusted by the follow-up time (up to 3–4 years following randomisation) — comparison of incidence in intervention *v* the control arm

cfDNA = cell-free DNA; CSO = cancer signal origin; NHS = National Health Service; PET-CT = positron emission tomography–computed tomography; UK = United Kingdom; US = United States.

* All cancers.

† Ovarian and endometrial cancers.

‡ Fifty types of cancer, including breast, colorectal and ovarian cancer.

## 4 Summary of practice points

**Table:**

	Practice points	
	Average risk individuals	High risk individuals
Screening for ovarian cancer	Screening for ovarian cancer with Ca125 and/or transvaginal ultrasound is not currently recommended for asymptomatic women at average population risk	Annual Ca125 and transvaginal ultrasound should not be undertaken for women at high risk Screening is not currently routinely recommended in women at high risk The potential role of interim surveillance every 4 months as a risk management strategy in women who prefer to defer or delay surgical prevention is currently being evaluated by NICE (UK)
Genetic testing	Women with high grade epithelial ovarian cancer should be offered panel genetic testing for ovarian cancer susceptibility genes *BRCA* testing should be offered to all Jewish individuals (unselected population testing), irrespective of family history, following informed consent Unselected general population-based genetic testing studies are being undertaken in Australia (DNA Screen)^78,80,81^ and the UK (PROTECT-C)^79^	Individuals with a strong family history of cancer should be referred for counselling and testing for an ovarian cancer susceptibility gene if the overall pre-test probability is > 10% Cascade testing can be offered to family members of individuals with a known pathogenic variant in an ovarian cancer susceptibility gene
Preventing ovarian cancer	Bilateral salpingo-oophorectomy for ovarian cancer prevention alone should not be undertaken in individuals at average population risk (1.3–2% lifetime risk of ovarian cancer)	RRSO is the most effective method to reduce ovarian cancer risk. It should be offered in pre-menopausal women with ≥ 4% lifetime risk of ovarian cancer or post-menopausal women with ≥ 5% lifetime risk of ovarian cancer RRSO may be undertaken in *BRCA1* carriers from age 35 years onwards, *BRCA2* carriers from age 40 years onwards, and in *RAD51C, RAD51D, PALB2* and *BRIP1* carriers from age 45 years onwards An early salpingectomy, followed by delayed oophorectomy (new two-stage surgical prevention procedure), is currently being evaluated in individuals at high risk and should only be undertaken as part of a research trial
	Opportunistic bilateral salpingectomy (OBS) may be offered at routine gynaecological surgery to all women who have completed their family following informed counselling of advantages and disadvantages	Provided no contraindication, women undergoing premature menopause following pre-menopausal RRSO should be offered HRT until 51 years (age of natural menopause) The HRT plan should be discussed and agreed before surgical prevention (RRSO)
	Ongoing trials are assessing the impact of OBS on patients’ reported outcomes. Prospective studies with long term follow-up are needed to assess the impact on cancer risk reduction and menopause	Patients are best managed with multidisciplinary team input in a specialist high risk clinic. They should receive evidence-based advice on HRT, symptom management, as well as specialist counselling and ongoing support to manage potential physical, emotional and long term health consequences In women with good prognosis triple negative breast cancer, short term HRT use on an individualised basis should be discussed with a breast oncologist and menopause specialist with a special interest in this area A SEE-FIM protocol should be used for histopathological assessment following RRSO Peritoneal washings should also be undertaken at RRSO

Ca125 = cancer antigen 125; HRT = hormone replacement therapy; NICE = National Institute for Health and Care Excellence; UK = United Kingdom; RRSO = risk-reducing salpingo-oophorectomy; SEE-FIM = Sectioning and Extensively Examining the Fimbria.
